# How Does the Basic Urban–Rural Medical Insurance Affect Resident Health Inequality? Evidence from China

**DOI:** 10.3390/healthcare13121455

**Published:** 2025-06-17

**Authors:** Xiaohong Pu, Riyun Hou, Sichang He, Weike Zhang

**Affiliations:** 1 School of Public Administration, Sichuan University, Chengdu 610065, China; puxiao028@163.com (X.P.); 2009020@hbmzu.edu.cn (R.H.); zhangwk@scu.edu.cn (W.Z.); 2 The Affiliated Hospital of Southwest Medical University, Luzhou 646000, China

**Keywords:** urban–rural resident basic medical insurance, health inequality, participants, concentration index

## Abstract

Background: Health inequality is seen as a challenge for implementing the Healthy China Strategy. This study analyzes the income-related health inequality among urban–rural resident basic medical insurance (URRBMI) participants. Methods: This study utilized data from the 2019 China Household Finance Survey (CHFS), and the concentration index (CI) was employed to estimate the effects of income-related health inequality on participants. Results: Our findings provide clear evidence that health inequality among participants has fluctuated—narrowing, widening, and then narrowing again—in the areas of the contribution, medical treatment, and reimbursement of URRBMI, respectively. Overall, the analysis indicates a widening of health inequality post-reimbursement, with results remaining consistent. A heterogeneity analysis shows that health inequality is most pronounced among women and those with less than a middle school education. Finally, our study reveals a pro-rich trend in the actual utilization of medical services among participants, with persistent disparities in outpatient and inpatient service usage even after standardization, further exacerbating income-related health inequality. Conclusions: We recommend that the URRBMI design take participants’ income levels into account, with policies favoring disadvantaged individuals to enhance their medical security, improve access to healthcare services, and ultimately reduce health inequality.

## 1. Introduction

Health inequality is a worldwide problem and is prevalent in both developed and developing countries [1,2,3]. Absolute levels of health inequality are rising in some countries [4], and they exist both between regions and between populations. In our study, health inequality is defined as the income-related health inequality of participants, emphasizing an inequality in health outcomes. Sustainable development goals also emphasize reducing inequality [5]. Eliminating health inequality related to socioeconomic status has become one of the key policy objectives of national health systems around the world [6,7]. Health inequality is an important manifestation of social inequality, and if it is too serious, it can slow down or even prevent the healthy development of society. Mooney argues that achieving health equality should be prioritized as the most important goal and that when there is a choice between efficiency and equality, achieving the goal of promoting health equality should also be prioritized [8]. Compared to other forms of inequality, health inequality is particularly concerning, because its existence directly threatens the basic well-being of individuals and can be an important trigger for other new social inequalities.

Health inequality related to socioeconomic status is a challenge that must be overcome to implement the Healthy China Strategy. China’s life expectancy has more than doubled from 35 years at the beginning of the country’s establishment to 79 years in 2024, and the health level of China’s residents has improved overall. However, improvements in the overall health of the population cannot compensate for health inequality problems, and some studies have revealed that the degree of health inequality in China is increasing [9].

Urban–rural resident basic medical insurance (URRBMI), as an important public policy for safeguarding the health of China’s residents [10], is an effective measure for intervening in health inequality [11]. However, in fact, equal access to participation does not mean equal benefits in terms of health outcomes [12]. From the perspective of social equity and justice, URRBMI should ensure that residents with different incomes can benefit equally from the same medical needs and should even focus on tilting the balance in favor of vulnerable residents to ensure that they are able to achieve a greater degree of improvement in their health status and thus reduce health inequality, which is also the correct value orientation that should be held by the URRBMI. As a social insurance system with a major bearing on the health protection of the entire population [13], if we can accurately recognize the role played by income in the URRBMI from the perspective of health inequality, the basis for the future reform and improvement of the URRBMI will be established. In general, conducting research on health inequality among participants in the URRBMI is highly theoretically and practically important.

This study makes the following contributions. First, according to the statistical results, the social medical insurance participation rate of residents is greater than 95%. Unlike existing studies, we estimate the income-related health inequality of participants rather than the effect of being insured or uninsured on health inequality. Second, we explored the heterogeneity of health inequality among participants from the perspectives of gender and education and offered some critical perspectives for improving health inequality in the future. Third, we discuss the pathways that influence health inequality among participants from the perspective of the standardization of medical service utilization.

### Literature Review

While medical insurance improves the financial accessibility of medical services for participants, whether it ultimately enhances their health status and reduces health inequality has become an important criterion for evaluating the effectiveness of the medical insurance system. The current research related to basic medical insurance and health inequality can be categorized into two main areas. The first area examines the impact of participation in basic medical insurance on health inequality among residents. They believe that the expansion of medical insurance coverage has significantly improved the health status of participants and helped reduce health inequality [14,15,16,17]. This could be the result of being able to access more high-quality medical services by enrolling in medical insurance [18,19,20]. However, some scholars have argued that the participation in medical insurance has not alleviated health inequality and that health inequality has tended to increase [21,22,23,24]. The second area focuses on the impact of the urban–rural integration policy of basic medical insurance on health inequality among residents. Sun et al. [25] reported that health inequality among residents with different incomes improved with increasing URRBMI. This finding is also consistent with the conclusions of Zhou et al., Bai et al., and He [26,27,28].

Many interesting results have been reported on the above issues, yet some gaps remain that need to be addressed. When all residents qualify for basic medical insurance, examining health inequality from the perspective of “participation or non-participation” in URRBMI is relatively superficial, and it is more important to pay attention to the impact of the level of protection of the URRBMI on the health inequality of the participants. However, less attention has been given to the impact of health inequality among participants in the framework of the URRBMI. Therefore, in the background of realizing the universal coverage of basic medical insurance in China, it is of greater theoretical and practical value to carry out research related to the health inequality of URRBMI participants.

## 2. Theoretical Analysis

Income rank changes lead to differences in the health level of the population, which is reflected in changes in health inequality, and income plays a vital role in health inequality [29,30,31]. The income hypothesis suggests that the income level of the population and the relative distribution of income among the population are important factors that influence the health of the population. URRBMI, which is closely related to the health of residents, has just undergone a profound system transformation and upgrading, and the changes in socioeconomic factors embedded in the transformation process undoubtedly have a deep impact on the health inequality of the participants. The URRBMI mainly consists of three parts: contribution, medical treatment, and reimbursement. Research shows that the health of low-income individuals is more sensitive to changes in income [32] and that improving the income status of low-income individuals has important health effects by helping to improve the distribution of health inequality in the population. URRBMI can help solve the problem of income-related health inequality among the participants by realizing “transfer payments” of income among the participants through the three segments of contribution, medical treatment, and reimbursement, which can increase the income level or improve the welfare status of the low-income participants.

This study explores income-related health inequality among participants; the three segments of contribution, medical treatment, and reimbursement imply that the income of the participants decreases, decreases, and increases, respectively. The three segments involved in URRBMI affect the income level of the participants, and the rank order of the participants’ income changes accordingly, thus affecting the distribution of health inequality among the participants. Therefore, this study intends to research health inequality among participants by focusing on the three segments of contribution, medical treatment, and reimbursement of URRBMI.

At present, URRBMI is based on a fixed contribution. However, the government departments implement categorized premium assistance policies for individuals who have difficulties paying, such as extremely poor individuals, low-income families with difficulties, disabled persons, etc. The financial department will bear part or all of their individual contribution, which enhances the payment capability of vulnerable participants and avoids the lack of medical protection for them for financial reasons. Furthermore, the demand for medical services for vulnerable participants, who are restricted by the price of medical services, will be further released, which may improve the quality of the utilization of medical services and reduce the probability of “having illnesses but not receiving medical treatment” and “minor illnesses being delayed to become major illnesses”, thus contributing to the improvement of their health conditions. As a result, the level of income-related health inequality among participants may decline in the contribution segment, so that it contributes to reducing health inequality among participants.

URRBMI has lowered the prices of medical services faced by all participants when they utilize medical treatment, thus enhancing their ability to consume medical services. As there are still significant differences in the ability of participants with different incomes to bear the costs of specific medical treatments, participants still need to bear part of the costs after seeking medical treatment, which affects the specific medical choices and behaviors of participants with different incomes when they fall ill, such as the type of medical services they choose, the level of medical institutions they choose to visit, and the level of treatment they receive. Low-income participants may choose outpatient treatment or lower-level medical institutions more often, shorten the length of their medical treatment, or simply give up treatment due to their income, which may result in less improvement in their health. However, higher-income participants—who face fewer financial constraints, have a wider range of choices for medical treatment, and a higher level of choice of medical institutions—are likely to experience greater health improvements, which may increase the degree of health inequality among participants with different incomes. As a result, the medical treatment segment may increase health inequality among participants.

For the predictable reimbursement of URRBMI, low-income participants can rely on medical insurance protection when they face health risks, thus reducing the motivation to give up medical treatment and providing strong support for health improvement [33]. The absolute income hypothesis suggests that the health status improves as the average income increases [34]. Outri et al. [35] reported that the health income elasticity, which decreases with an increasing income, is favorable for reducing health inequality if the income increases. The reimbursement of URRBMI compensates for the expenses of the participants for medical treatment and increases the income of all participants, and the health status of the participants improves after they receive reimbursements for medical treatment. However, there is a marginally decreasing phenomenon and heterogeneity in the degree of the improvement in their health. That is, although the health levels of higher-income participants will also improve, their health status is already relatively good to begin with, so the marginal improvement in health will have little effect. In contrast, reimbursement can significantly increase the ability of vulnerable participants to access medical resources and health services. Thus, their health improvement outcomes may be more significant. Ultimately, this differential health improvement effect contributes to a reduction in the level of health inequality across participants and can help reduce health inequality.

## 3. Materials and Methods

### 3.1. Materials

#### 3.1.1. Data Source

The data were collected from the China Household Finance Survey in 2019 (CHFS 2019). The CHFS provides comprehensive national survey data [36,37], which covers not only basic medical insurance but also areas such as health status and health risks. A total of 107,008 respondents were recruited from the CHFS 2019. The data include detailed demographic, health, economic, and medical service utilization data. For the purpose of this study, the sample was selected according to the following criteria: to retain the residents who only participated in the URRBMI and do not participate in other basic medical insurance. Additionally, the invalid samples with missing key information, including gender, age, education years, marital status, and region, were removed. Finally, 64,786 respondents were selected from the sample after data cleaning.

#### 3.1.2. Variable Definitions

Measuring health inequality requires information about individual-level health and income status [38]. As an individual’s overall perception of health status, self-rated health (SRH) is a valid composite indicator that comprehensively reflects the multidimensional and holistic nature of health. It is now widely used to measure health inequality in the population [39,40,41,42,43,44,45]. Therefore, this study also uses participants’ SRH to measure health inequality. The SRH is measured by the 5-point Likert scale in the CHFS 2019 questionnaire, and we constructed an ordinal variable consisting of 5 levels. The higher the score was, the better the participants’ health status.

To accurately estimate the health inequality of URRBMI, we controlled several variables as follows: gender, age, education years, marital status, and region.

### 3.2. Methods

The concentration index (CI) can be used to measure the socioeconomic status of variables [46,47,48,49]. A detailed portrayal of the distribution of health under the socioeconomic status of an operation is simple and straightforward. Therefore, CI is preferred by most scholars and has become an important method for measuring the degree of inequality in health [50,51]. Compared with other methods, the CI is a more comprehensive and specific measure of health inequality, with greater accuracy and sensitivity. CI has been widely utilized in measuring income-related health inequality across countries [52,53], which summarizes how cumulative shares of health are associated with cumulative shares of the population ranked by income [54]. Bommier and Stecklov noted that the CI is a more reasonable measure of inequality than the inequality index derived from the social welfare function [55].

The CI requires that health indicators be binary dummy or continuous variables and cannot be ordered variables. Therefore, this study refers to the research of Van Doorslaer and Jones [56] and uses the Ordered Probit model to transform SRH indicators with ordered variables into continuous variables in the interval of [0, 1]. The specific operation idea of the method is as follows:

*H_i_* = *α*_0_ + *α*_1_*Insurance* + *α*_2_X_i_ + *ε_i_*, ε~N(0, 1) (1)

where *H_i_* represents the SRH level of the participant, *Insurance* represents the income status of the participant at different stages, *X_i_* represents a series of control variables, *α*_0,_ *α*_1,_ and *α*_2_ represent the corresponding coefficients, and *ε_i_* represents the random error term.

The individual SRH score for the continuous variable was subsequently calculated according to Equation (1), which takes values in the range of (−∞,+∞), and then Equation (2) was used to assign it to a value in the interval [0, 1].

(2) $${\mathrm{SaH}}_{i}=\frac{H_{i}-\min(H_{i})}{\max\left(H_{i}\right)-\min(H_{i})}$$

where *SaH_i_* represents the SRH level of the participant after the switch, with *max*(*H_i_*) representing the best SRH level after the switch and *min*(*H_i_*) indicating the lowest SRH level after the switch.

Next, we calculate the health CI. Drawing on Wagstaff et al. [57], the health CI is calculated as

(3) $$\mathrm{CI}=\frac{2}{H}cov\left(S{aH}_{i},I_{i}\right)=\frac{2}{n*H}\sum_{i=1}^{n}{SaH}_{i}I_{i}-1$$

*CI* was adopted to analyze and compare health inequality. *H* represents the average health level of the participants; *I_i_* denotes the rank of sample *i* in terms of the quantile value in the ranking according to income status from lowest to highest, obtained by (*i* − 0.5)/*n*; and *n* denotes the number of sample observations. *SaH_i_* represents the transformed SRH, with larger values representing better health for the individual. The *CI* lies between [−1 and +1], where 0 indicates no income-related health inequality of participants with URRBMI, and there is a positive (negative) score when there are income-related health inequality participants with URRBMI favoring the rich (poor). In this study, all analyses were conducted using the statistical software StataSE-64 (version 15.1, StataCorp LLC, College Station, TX, USA), and the statistical significance level was set at *p* < 0.01. Descriptive statistics for SRH were reported as frequency (%) and means.

## 4. Results

### 4.1. Descriptive Statistics

Table 1 shows the SRH distributions of the participants’ income quintile groups. The second column shows that with the gradual increase in income, the proportion of participants whose SRH is very poor tends to decrease. The proportion of participants with very poor SRH was the highest in the low-income group, at 4.87%, whereas the proportion of participants with very poor SRH was the lowest in the high-income group, at only 1.53%. The fifth and sixth columns show that as the income increases, the total proportion of participants whose SRH is good or very good increases gradually, and the total proportion of participants in the high-income group whose SRH is good or very good is higher than that of participants in the low-income group, indicating that the higher the income of the participants is, the better their SRH is. Similarly, the converted SRH score consistently revealed that the SRH score increased gradually with income, with participants in the high-income group having a health score that was 0.0349 points higher than the health score of the low-income group. Overall, there was income-related health inequality among the participants.

### 4.2. Health Inequality

This section focuses on the effect sizes between income variations at the three segments of participants’ contribution, medical treatment, and reimbursement and the health inequality among the participants. First, the initial income is defined as the per capita household income of the participant, and then the initial income-related health inequality of the participants is measured. The changes in health inequality among participants in the three segments of contribution, medical treatment, and reimbursement were then calculated and compared to identify the impact of URRBMI on health inequality among participants. We use Δ*_total_* to denote the difference in health inequality among the participants at their initial income level and the health inequality after reimbursement. If Δ*_total_* is negative, it indicates that URRBMI has an inverse moderating effect on health inequality among the participants. In contrast, it has a positive moderating effect on health inequality among the participants.

Table 2 shows the changes in health inequality for participants across the segments. First, the CI of participants’ initial income is 0.0754, which indicates that there is a “pro-rich” health inequality among participants. Second, the CI of 0.0752 for participants’ contribution indicates that there is still a “pro-rich” health inequality in participants’ contributions. Compared with the CI of the initial income, the difference is positive, which indicates that participants’ health inequality is positively moderated in the contribution segment, meaning that after paying the premium, the overall level of health inequality among them decreases. Third, the CI of 0.0821 for participants’ medical treatment suggests that there is a “pro-rich” health inequality in participants’ medical treatment. Compared with the CI in the contribution segment, the difference is negative, which indicates that participants have a negative mediating effect on health inequality during this stage, meaning that the level of health inequality among them has increased. Fourth, the CI for participants in reimbursement is 0.0785, showing that there is a “pro-rich” health inequality in reimbursement for participants. Compared with the CI of the medical treatment segment, the difference is positive, indicating that health inequality is reduced when participants receive reimbursements, meaning that the reimbursement segment positively moderates health inequality.

In general, the CI of participants’ initial income is 0.0754, whereas the CI after the reimbursement is 0.0785, and the value of Δ*_total_* is −0.0032, which implies that participants’ health inequality has instead widened after the contribution, medical treatment, and reimbursement. In general, high-income participants possess a higher socioeconomic status and have the ability to obtain better quality health resources, resulting in relatively better medical protection levels. This reduces the health risks for high-income participants and generally leads to better health outcomes than for low-income participants, thus creating the phenomenon of health-income stratification. In terms of the income groups, Δ*_total_* is positive for both the upper-middle-income group and the high-income group, and there is a decrease in the level of health inequality in these two groups. In contrast, the CI after the reimbursement for the low-income, lower-middle-income, and middle-income groups is greater than the CI for the initial income, and the absolute value of Δ*_total_* (|−0.0605| > |−0.0038| > |−0.0037|) gradually decreases as income increases, which indicates that the degree of health inequality has widened for these three income groups.

### 4.3. Robustness Test

This study utilized two different methodologies to conduct a robustness test.

#### 4.3.1. Robustness Tests Based on the EI

In this study, we first use the Erreygers concentration index (EI) method for robustness testing. In 2009, Erreygers formalized a modified concentration index method, later named the Erreygers concentration index [58]. Since then, Erreygers, Wagstaff et al., and Lambert et al. have explored all aspects of the applicability of the EI, and the EI has been continuously improved [59,60,61]. The EI is also widely used in measuring health inequality because it is more scientific and accurate in calculating the level of inequality for continuous bounded effect variables. In the studies of He [28] and Allanson et al. [62], the EI was further used to measure health inequality among participants to conduct a robustness test of the main findings above. The formula is as follows:

(4) $$EI(h|y)=\frac{1}{n}\sum_{i=1}^{n}\left[\frac{4h_{i}}{\left(h_{\max}-h_{\min}\right)}(2R_{i}-1)\right]$$

where *h_i_* indicates the participant’s SRH level. *h_max_* represents the best SRH level, and *h_min_* represents the lowest SRH level. *y* is the participant’s initial income, and *R_i_* is the rank of the initial income in the total sample. The EI takes values in the range of [−1, 1], and when it takes the value of [−1, 0), it indicates that health inequality favors low-income participants. When it takes the value of (0, 1], it indicates that health inequality favors high-income participants. And when it takes the value of 0, it indicates that there is no health inequality. The greater the absolute value of the EI is, the greater the degree of the health inequality.

Table 3 reports the EI for participants in the initial income, contribution, medical treatment, and reimbursement segments. The EI for participants as a whole is positive for the initial income, contribution, medical treatment, and reimbursement, suggesting that there is a “pro-rich” health inequality among participants as a whole. In general, the EIs after the reimbursement are greater than the initial EI (0.1681 > 0.0281). This suggests that URRBMI increases health inequality and again confirms the robustness of the previous conclusions.

#### 4.3.2. Robustness Tests Based on Health Relative Deprivation

Stouffer et al. developed the theory of relative deprivation (*RD*) in 1949. That is, the worse the health of the population within a group is, the greater the degree of relative deprivation suffered in the accumulation of health disadvantages. In Fukushige’s study, health inequality among participants with different incomes was measured by constructing an index of relative health deprivation [63]. We use participants’ SRH as a variable to measure relative health deprivation. Within a group, the worse a participant’s SRH is, the greater the health disadvantage and the more obvious the relative felt deprivation of health, meaning that the degree of health inequality is greater. According to Kakwani’s definition, assuming that *X* represents a cohort with a sample size of *n*, the participants’ SRH scores are ranked in ascending order to obtain the SRH distribution of all participants, *X =* (*X_1_, X_2_,……, X_n−1_, X_n_*), where *X_1_ ≤ X_2_ ≤ …… ≤ X_n__−1_ ≤ X_n_*. By comparing the SRH of the *i* participant *X_i_* to the SRH of the *j* participant *X_j_*, the *RD* of this participant can be expressed as follows:

(5) $$RD(x_{j},x_{i})=\left\{\begin{array}{r} x_{j}-x_{i},\;\mathrm{if}(x_{j}>x_{i})\\ 0,\;\mathrm{if}(x_{j}\leq x_{i}) \end{array}\right\}$$

where the relative deprivation of the health of the *i* participant (*x_j_*_,_ *x_i_*) implies the relative deprivation of *x_j_* to *x_i_*, and by summing RD (*x_j_*_,_ *x_i_*) over *j* and dividing by the mean value of the participant’s SRH, the average relative deprivation of the SRH of the *i* participant can be expressed as follows:

(6) $$RD\left(x_{i}\right)=\frac{1}{nu_{x}}\sum_{j=1}^{n}RD(x_{j}-x_{i})=\frac{1}{nu_{x}}(\sum_{x_{j}>x_{i},\;x_{j}\in X}x_{j}-\sum_{x_{j}>x_{i},\;x_{j}\in X}x_{i})$$

By further decomposing Equation (6), the average health-related deprivation of participants can be transformed as follows:

(7) $$RD(xi)=\frac{1}{nux}\left(n_{\mathrm{xi}}^{+}\times u_{xi}^{+}-n_{xi}^{+}\times xi\right)=\frac{1}{ux}r_{xi}^{+}\left(u_{xi}^{+}-xi\right)$$

where *n* is the sample size, *u_x_* is the mean value of the SRH for all participants in the group, $n_{xi}^{+}$ is the number of participants in the group whose SRH exceeds *x_i_*, $r_{xi}^{+}$ is the number of samples in the group whose SRH exceeds *x_i_* as a percentage of the total sample, and $u_{xi}^{+}$ is the mean value of SRH for samples whose SRH exceeds *x_i_* in the group.

Table 4 reports the results of the regression for participants based on relative health deprivation. According to columns (1) and (2), regardless of controlling for other variables, the relative health deprivation is lower for participants in the middle-income, upper-middle-income, and high-income groups than for participants in the low-income group, all of which are negatively significant at the 1% level, with low-income participants having a significantly greater relative health deprivation than participants in the other income groups. The absolute value of the coefficient of the relative health deprivation increases as the income level increases, so the relative health deprivation of participants in the low-income group is greater. It indicates that participants in the high-income group have a greater relative health deprivation than the participants in the low-income group and that there are indeed health inequalities among the participants. The outcome is in accordance with the previous results, suggesting that the results remain highly robust.

### 4.4. Heterogeneity Analysis

We also explored the heterogeneity of the health inequality among participants from the perspectives of gender and education. The estimation results are shown in Table 5. Rows (1) and (2) are the results of gender, and Rows (3) to (6) are the results of education.

Generally, the CI is positive for both male and female participants, indicating a “pro-rich” health inequality for both males and females. The CI of male participants in all segments is smaller than that of female participants, which shows that there are some gender differences in health inequality among the participants and that health inequality is greater among female participants. In addition, the positive value of Δ*_total_* for female participants and the negative value of Δ*_total_* for male participants indicate that the health inequality of female participants has declined and that the health inequality of male participants has widened after being adjusted by the URRBMI.

The CIs by educational level are all positive, and there is a “pro-rich” health inequality. As the education level increases, the overall trend of the CI of each segment gradually decreases, especially in the medical treatment and reimbursement segments, which are more obvious. The CI of the education level above high school is the smallest, and that of the education level below middle school is the largest, indicating that there is a difference in the education level of the health inequality of the participants and that the higher the education level is, the lower the degree of the health inequality of the participants is. In fact, the Δ*_total_* values of the participants in different educational groups are all negative, which shows that the health inequality of the participants has been expanded after the adjustment of the URRBMI.

### 4.5. Medical Service Utilization

Equity in medical service utilization is an important way to achieve health equality, which is highly practical for improving health inequality among participants and promoting the development of a healthy China. URRBMI needs to influence the health status of participants with the help of medical service utilization [64], which is essential to maintain and promote the health of participants. Only when everyone has equal access to basic medical services can the health inequality among participants be minimized or even eliminated. In general, all people should have access to the same medical service utilization as long as the participants are in need, regardless of their financial ability to pay [65]. In reality, however, the income level affects the utilization of medical services when a participant is sick, with higher-income participants having sufficient ability to pay to receive more medical services. However, less medical service utilization consumes most of the income of lower-income participants, and lower-income participants are in poorer health, resulting in health inequality.

The ideal state of URRBMI should be that all participants can have approximately the same medical service utilization when they have the same medical service needs. It is necessary and important to standardize the need for medical services among participants before evaluating the equity of their medical service utilization. This section explores post-standardization medical service utilization among participants from a horizontal equity perspective, analyzing whether participants with the same medical service needs receive equal medical service utilization. Horizontal equity means that regardless of the socioeconomic status of participants, they should receive the same level of medical service utilization if they have the same medical service needs. The factors affecting medical service utilization among the participants can be classified into two categories, needs and no needs, with “needs” factors such as disease severity, age, gender, etc., and “no needs” factors such as income, education level, marriage, etc. Theoretically, the medical service utilization among the participants should be influenced only by the factors of medical service needs, and the inequality of medical service utilization caused by “needs” factors is reasonable and acceptable. If there are interventions from other no needs factors, the inequality of the medical service utilization among participants can be considered to exist [66].

Referring to Pulok et al.’s study [67], we chose the indirect standardized method, which is still widely used, to measure the degree of equality in medical service utilization among participants. The specific steps are as follows [68]:

(1) The first step is to estimate the actual medical service utilization of the participants. The following regression equation is established:
  *Y_i_* = *α* + *β_i_X_i_* + *γ_i_Z_i_* + *ε_i_* (8)
  where *Y_i_* is the medical service utilization of the *i* participant; *X_i_* represents the “needs” factors affecting the medical service utilization among participants, such as gender, age, and SRH. *Z_i_* is the “no needs” factors affecting medical service utilization among participants, such as their income, education level, marital status, urban/rural status, and region. *β_i_* and *γ_i_* are the regression coefficients of the corresponding variables. *ε* represents the residuals, indicating the differences not explained by the regression equation.
(2) Then, we estimate the amount of the medical service utilization of the participants influenced by “needs” factors, namely, the expected medical service utilization. Expected medical service utilization refers to the medical service utilization predicted by the “needs” factor according to the model. The regression equation is as follows:
  (9) $${\hat{Y}}_{i}=\;\hat{\alpha }+{\hat{\beta }}_{i}X_{i}+{\;\hat{\gamma }}_{i}{\bar{Ζ}}_{i}+\varepsilon$$
  where ${\hat{Y}}_{i}$ is the predicted participant medical service utilization for the “needs” factor, and where ${\bar{Z}}_{i}$ is the mean value of the *i* “no needs” factor.
(3) Finally, we calculate the medical service utilization of the participants after standardizing the “needs” factor. This reflects the amount of medical service utilization determined by socioeconomic factors among participants with the same need for medical services. The equation is as follows:
  (10) $$Y_{s}=Y_{i}-{\hat{Y}}_{i}+\bar{Y}$$
  where *Y_s_* is the participant’s standardized medical service utilization, in which $\bar{Y}$ is the participant’s actual mean value of medical service utilization.

Table 6 presents the distribution of the outpatient and inpatient medical service utilization among participants grouped by income quintiles.

There is a significant difference in the actual utilization of outpatient services among participants in different income groups, with the lowest actual utilization of outpatient services among participants in the low-income group, which is only 82.76% of the expected need for outpatient services, indicating that the actual demand for outpatient service utilization by low-income participants with poorer health conditions has not yet been met. In contrast, the actual outpatient service utilization was greater than the expected need for outpatient service utilization for the remaining income groups of participants, indicating that the tendency to overuse outpatient services is more apparent among participants with higher incomes. After standardizing the utilization of outpatient medical services, the difference in the utilization of outpatient services among participants with different incomes increases, indicating that when participants in each income group have the same health status, the utilization of outpatient services among participants in the high-income group is still significantly greater than that among participants in the low-income group and that fairness in the utilization of outpatient services for the same needs has not yet been achieved. Low-income participants with poor health should need more medical services to improve their health, but this is not the case, and health inequalities among participants are aggravated by inequality in the outpatient service utilization among participants with different incomes.

The actual utilization of inpatient services among participants in different income groups showed a stepwise change. That is, participants in the lowest income group had the lowest level of actual utilization of inpatient services (0.270), and participants in the highest income group had the highest level of the actual utilization of inpatient services (0.938), which suggests that participants in the higher income group, who are in better health, instead utilize more inpatient health services, which could be the source of health inequality among participants. The actual inpatient service utilization for participants in the low-income group and participants in the lower-middle-income group (0.270 and 0.646) was much lower than the expected need for inpatient service utilization (0.743 and 0.735), indicating that the actual inpatient service utilization is insufficient for participants in the low-income group and participants in the lower-middle-income group. The actual utilization of inpatient services for participants in the middle-income group, upper-middle-income group, and high-income group is greater than the expected need for inpatient services, which are 107.48%, 129.39%, and 149.60% of the expected need for inpatient services, respectively, showing the characteristic of “low need, high utilization”. After standardizing the inpatient service utilization, participants with higher incomes had a greater inpatient service utilization, indicating that inequalities in the inpatient service utilization among participants still existed after standardization.

## 5. Discussion

### 5.1. Contribution and Reimbursement Help Reduce Health Inequality

In this study, we find high health inequality among participants in terms of contribution, medical treatment, and reimbursement. In addition, the contribution and reimbursement segments relatively reduce health inequality for participants. The URRBMI provides financial subsidies for the individual contributions of some disadvantaged participants, which, to a certain extent, resolves the worries of disadvantaged participants about their medical insurance contributions. As a result, income-related health inequality among participants declines in the contribution segment, and it positively moderates the health inequality among participants. This is consistent with Hu et al.’s findings that subsidies contribute to equity in benefit incidences [69]. This study also shows that reimbursement compensates disadvantaged participants to a certain extent for their medical expenditures, reduces the burden of medical costs on disadvantaged participants, and has a greater impact on health improvement in terms of predictable reimbursement. As disadvantaged participants can rely on medical insurance to reduce their exposure to health risks, the incentives to abandon medical care after illnesses can be reduced. Ultimately, this differential health improvement reduces health inequality.

Stabilizing participation in the insurance system, as the entrance point for the implementation of URRBMI rights and benefits, is both the key to the continued expansion of the medical insurance system coverage and an important precondition for the promotion of fair access to medical insurance benefits for participants. Many countries around the world have expanded basic medical insurance coverage in the form of compulsory participation. Therefore, we should improve the design of the URRBMI policy. It is proposed to change the form of voluntary participation in our country to compulsory participation to realize universal coverage and accelerate the establishment of a long-term mechanism for stable participation in URRBMI. In particular, at present, the URRBMI is characterized by a fixed contribution and the lack of a unified and standardized mechanism for financing the growth and dynamic adjustment of insurance rates, which is contrary to the basic principle that the obligation to pay contributions to the social insurance system should be linked to the level of the income of the participants. Therefore, it is recommended that, in accordance with the principle of affordability, a contribution mechanism be implemented that is related to the income level of the participants [70] and that appropriate contribution rates be set to form a mechanism for stabilizing and moderating the growth of contributions. In this way, the contribution burden on low-income participants will be reduced, their ability to provide health protection will be enhanced, and the health inequality among participants will be reduced. In addition, the effect of relying solely on URRBMI on improving health inequality among participants may not be obvious. The multilevel medical insurance system, because of its large reimbursement scope and strong protection, greatly shares the protection pressure of basic medical insurance and better meets the diverse medical protection needs of participants. Therefore, we should focus on strengthening the collaborative governance capacity of multilevel medical insurance to meet the diverse health protection needs of participants, to effectively increase their level of health protection and reduce health inequality.

### 5.2. Health Inequality Differs by Gender and Education

The results of this study show that the CI of male participants in all segments is smaller than that of female participants, which indicates that health inequality is greater among female participants and that there are gender differences in health inequality among participants. This may be related to the fact that in the course of China’s economic development, women not only have to work but also have to invest more time and energy in family labor. Coupled with the long-established “patriarchal” ideology, this has resulted in women facing greater health inequality than men do. At the same time, the difference between the CI of male and female participants becomes progressively smaller after they have gone through the contribution, medical treatment, and reimbursement stages. That is, the health inequality gap between the genders gradually decreases. In addition, the overall trend of the CI for each segment gradually decreases as the education level increases, indicating that there is heterogeneity in the health inequality of the participants in terms of education. That is, the higher the education level of the participant is, the lower the level of health inequality.

Owing to the heterogeneity of health inequalities by gender, the degree of health inequality is greater among female participants. To narrow the gender–health gap among participants, it is necessary to pay more attention to the health status of female participants in the future, strengthen the monitoring of the health level of female participants, and pay close attention to the factors affecting the health inequality of female participants to achieve the goal of gradually narrowing the inequality in the health of female participants. Moreover, academic qualifications are a protective factor for improving health inequalities among participants. Education is an integral part of socioeconomic status, enabling people to make better use of health information [6]. Health inequalities can be reduced by increasing the educational level of participants. Therefore, the academic education of participants should be gradually strengthened, and the overall level of education should be improved to reduce the gap in health inequality among participants. Furthermore, increasing participants’ exposure to medical knowledge could be implemented to reduce health inequality.

### 5.3. Inequality in the Utilization of Medical Services by Participants

The URRBMI aims to improve health by guaranteeing equal access to medical services for the participants. There is no necessary link between an increase in the supply of medical services and equal access to them. The key is to determine who will benefit more from the utilization of medical services. This study revealed that low-income participants in poorer health use medical services less and that low-income participants’ actual medical service utilization is significantly lower than their expected medical service utilization. After the standardization of medical services, large differences remained. Yan et al.’s and Pan et al.’s studies also revealed that inpatient service utilization was pro-rich [71,72]. The URRBMI shares the costs of medical services among participants with different incomes, and out-of-pocket costs for medical services have declined, which tends to increase the demand for medical services among participants. However, low-income participants are still limited by their income levels and do not utilize medical services to the same extent as higher-income participants do. Therefore, the financial burden of low-income participants may not actually be reduced, and the health inequality gap widens in the medical treatment segment.

The URRBMI promotes fair and efficient access to basic medical services for participants to realize the policy objective of improving their health. The equitable utilization of medical services is an important channel for improving the health status of participants. Given the background of the basic realization of universal medical insurance, the design of future medical insurance policies needs to further consider the heterogeneity of participants, adopting the principle of “uniform but differentiated” to improve the precision of the implementation of basic medical insurance policies [73] and to more accurately meet the health protection needs of different participants. The idea of stepped reimbursement can be adopted, and differentiated compensation measures, such as lowering the starting line and increasing the actual reimbursement rate [74,75], can be taken for some specific groups of people with high medical service needs and low medical service utilization to improve the actual degree of benefits from medical insurance for disadvantaged participants, enhance their ability to utilize medical services, and ultimately maximize health rights and minimize health inequality.

## 6. Limitations

Despite its contributions, this article has the following limitations, and further research is needed. First, although SRH is the most important variable in predicting the circumstances of participants’ subsequent deaths, the measurement of health inequality is based on the SRH of the participants, which is somewhat subjective. Future studies could minimize bias by adding objective health measures. Meanwhile, although scholars often use ordered probit models to convert SRH, there may still be limitations such as pseudo-precision and non-linearity, and multiple methods can be used in future studies for comparative studies to further enhance the credibility of the interpretations. Second, we did not consider long-term changes in participant health inequality, and information on the long-term dynamic effects on participant health inequality could continue to be strengthened in future research. Third, this article discusses the inequality in medical service utilization, but, limited by time and effort, it does not provide a deep dive into the influencing factors behind it, which makes it an important direction for continuing in-depth research in the future.

## 7. Conclusions

Eliminating health inequalities has been one of the most important policies of public health departments in various countries. Using CHFS data from 2019, this study analyzed the health inequality among participants. We have come to the following conclusions.

First, the health inequality among participants narrowed, widened, and narrowed in the contribution, medical treatment, and reimbursement segments, respectively. And widened the most in the medical treatment segment. In terms of the overall moderating effect, it suggests that post-reimbursement, health inequality among participants has widened and that the results are robust. Second, is the heterogeneity in health inequality among participants in terms of gender and education. The results show that health inequality is highest among women and among participants with less than a middle school education. Furthermore, the actual utilization of medical services among all participants has a rich inequality trend, the actual outpatient and inpatient medical service utilization of lower-income participants with poorer health is lower than their corresponding expected outpatient and inpatient medical service utilization, and the inequality in the outpatient and inpatient medical service utilization among participants still exists after standardization, which increases the income-related health inequality among participants. As a whole, it has been proven that higher-income participants use more medical services than lower-income participants, which is an important cause of health inequality among participants, and that URRBMI contribution and reimbursement policies have been equalized, whereas the utilization of medical services and the degree of health improvement have not been equalized.

## Figures and Tables

**Table 1:** Health distribution of income quintiles of participants.

Groups	Initial SRH (%)					Converted SRH Score Means
	Very Bad	Bad	So So	Good	Very Good	
Total	5.16	17.88	40.17	26.07	10.72	0.0908
Low-income group	4.87	18.23	43.03	24.49	9.39	0.0772
Lower-middle-income group	4.86	17.09	43.32	25.79	8.95	0.0821
Middle-income group	3.45	16.32	41.48	27.43	11.31	0.0892
Upper-middle-income group	2.87	12.46	41.16	30.43	13.08	0.0933
High-income group	1.53	9.52	38.45	33.92	16.58	0.1121

**Table 2:** Concentration index distribution of participants across segments.

Groups	Initial Segment	Contribution Segment	Medical Treatment Segment	Reimbursement Segment	Δ*_total_*
Total	0.0754 ***	0.0752 ***	0.0821 ***	0.0785 ***	−0.0032
Low-income group	−0.0115 ***	−0.0117 ***	0.0562 ***	0.0490 ***	−0.0605
Lower-middle-income group	0.0154 ***	0.0154 ***	0.0175 ***	0.0192 ***	−0.0038
Middle-income group	0.0097 ***	0.0081 ***	0.0127 ***	0.0135 ***	−0.0037
Upper-middle-income group	0.0178 ***	0.0174 ***	0.0120 ***	0.0121 ***	0.0057
High-income group	0.0706 ***	0.0710 ***	0.0281 ***	0.0291 ***	0.0415

*** *p* < 0.01.

**Table 3:** EI distribution of participants across segments.

Groups	Initial Segment	Contribution Segment	Medical Treatment Segment	Reimbursement Segment
Total	0.0281 ***	0.0281 ***	0.1772 ***	0.1681 ***
Low-income group	−0.0037 ***	−0.0037 ***	0.0943 ***	0.0829 ***
Lower-middle-income group	0.0052 ***	0.0052 ***	0.0352 ***	0.0384 ***
Middle-income group	0.0036 ***	0.0030 ***	0.0281 ***	0.0293 ***
Upper-middle-income group	0.0068 ***	0.0067 ***	0.0280 ***	0.0279 ***
High-income group	0.0324 ***	0.0327 ***	0.0719 ***	0.0737 ***

*** *p* < 0.01.

**Table 4:** Regression results for relative health deprivation among participants with different incomes.

Variables	(1)	(2)
	*RD*	*RD*
Low-income group(reference)		
Lower-middle-income group	−0.00325	−0.00237
	(0.00239)	(0.00232)
Middle-income group	−0.0137 ***	−0.00735 ***
	(0.00235)	(0.00229)
Upper-middle-income group	−0.0345 ***	−0.0256 ***
	(0.00234)	(0.00229)
High-income group	−0.0611 ***	−0.0451 ***
	(0.00233)	(0.00233)
Control variables	*NO*	*YES*
Constant	0.185 ***	0.0928 ***
	(0.00184)	(0.00723)

*** *p* < 0.01, standard errors are in parentheses.

**Table 5:** Estimation results of heterogeneous analysis.

Groups		Initial Segment	Contribution Segment	Medical Treatment Segment	Reimbursement Segment
gender	(1) Male	0.0734 ***	0.0733 ***	0.0807 ***	0.0775 ***
	(2) Female	0.1298 ***	0.1295 ***	0.1010 ***	0.0974 ***
education	(3) Below middle school	0.0367 ***	0.0365 ***	0.0786 ***	0.0727 ***
	(4) Middle school	0.0290 ***	0.0289 ***	0.0573 ***	0.0534 ***
	(5) High school	0.0363 ***	0.0361 ***	0.0533 ***	0.0498 ***
	(6) High school and above	0.0262 ***	0.0261 ***	0.0364 ***	0.0337 ***

*** *p* < 0.01.

**Table 6:** Distribution of medical service utilization by participants.

Groups	Outpatient			Inpatient		
	Actual Utilization	Anticipated Need	Need to Standardize	Actual Utilization	Anticipated Need	Need to Standardize
Low-income group	3.724	4.500	3.618	0.270	0.743	0.222
Lower-middle-income group	4.521	4.480	4.435	0.646	0.735	0.607
Middle-income group	4.590	4.398	4.587	0.747	0.695	0.748
Upper-middle-income group	4.584	4.355	4.624	0.876	0.677	0.895
High-income group	4.552	4.239	4.708	0.938	0.627	1.006

## Data Availability

The original data are available at https://chfs.swufe.edu.cn/sjzx/sjsq.htm (accessed on 29 July 2024).
